# Identifying Key Genes and Functionally Enriched Pathways of Diverse Adipose Tissue Types in Cattle

**DOI:** 10.3389/fgene.2022.790690

**Published:** 2022-02-14

**Authors:** Cuili Pan, Chaoyun Yang, Shuzhe Wang, Yun Ma

**Affiliations:** ^1^ School of Agriculture, Ningxia University, Yinchuan, China; ^2^ Key Laboratory of Ruminant Molecular and Cellular Breeding, Ningxia Hui Autonomous Region, Ningxia University, Yinchuan, China

**Keywords:** adipose tissue, hub gene, biological process, weighted gene co-expression network analysis (WGCNA), cattle

## Abstract

**Background:** Fat is a tissue that not just stores energy and plays a protective role; it is also a vital endocrine organ that generates and integrates signals to influence metabolism. Meanwhile, the excessive accumulation of lipids in adipose tissue can lead to metabolic disturbance and diseases. To date, the complicated molecular mechanisms of bovine adipose tissue are still unknown. This study aimed to identify key genes and functionally enriched pathways in various adipose tissue types.

**Results:** The RNAseq data of 264 samples were downloaded from Gene Expression Omnibus (GEO) and analyzed by weighted gene co-expression network analysis (WGCNA). We identified 19 modules that significantly associated with at least one adipose tissue type. The brown module from GSE39618 was most closely associated with intramuscular fat tissue, which contained 550 genes. These genes were significantly enriched in pathways that related to inflammation and disease, such as TNF signaling pathway, IL-17 signaling pathway, and NF-kappa B signaling pathway. The pink module (GSE39618) that contained 58 genes was most closely associated with omental fat tissue. The turquoise (GSE39618), blue (GSE116775), and yellow (GSE65125) module were most closely associated with subcutaneous fat tissue. Genes in these modules were significantly enriched in pathways related to fat metabolism, such as the PPAR signaling pathway, fatty acid metabolism and PI3K-Akt signaling pathway. At last, key genes for intramuscular fat (*PTGS2* and *IL6*), omental fat (*ARHGEF5* and *WT1*), and subcutaneous fat (*KIT*, *QR6Q1*, *PKD2L1*, etc.) were obtained and verified. In addition, it was found that *IL10* and *VCAM1* might be potential genes to distinguish adipose and muscle.

**Conclusion:** The study applied WGCNA to generate a landscape of adipose tissue and provide a basis for identifying potential pathways and hub genes of different adipose tissue types.

## Introduction

Adipose tissue exists within multiple anatomical positions where it plays a role in controlling energy expenditure and regulating many metabolic processes (Cinti, 2005; McGown et al., 2014). It is usually considered as a major active endocrine organ that secretes adipokines, which can act locally or reach distant organs through the systemic circulation to exert a wide range of biological actions, including regulating food intake and body weight, insulin sensitivity, or inflammation (Smitka and Maresova, 2015). Lipids, such as triglycerides (TGs), excessively accumulated in internal adipose tissue can also lead to metabolic disturbance and diseases (insulin resistance, fibrosis, dyslipidemia, and cancer) (Cinti, 2012).

Previous studies have shown that adipose tissue from different anatomical positions usually exhibits different metabolic functions and regulatory mechanisms. Primarily, it has been studied in-depth in the regulation of disease. For instance, the study in gastric cancer patients with cachexia showed that subcutaneous (not visceral) adipose tissue could be a marker for prognosis (Han et al., 2021). Ectopic accumulation of visceral adipose was positively associated with adverse cardiometabolic consequences, while gluteal-femoral adipose accumulation was negatively correlated with these adverse complications, and abdominal subcutaneous adipose tended to show a more neutral association (Sam, 2018). Furthermore, the subcutaneous depots protected systemic glucose homeostasis and proximal muscle from metabolic dysregulation, and removal of them likely leaded to glucose intolerance because of the reduction of storage space for glucose and/or lipids (Booth et al., 2018). The increase of intramuscular adipose tissue, which could be promoted by FGF-2-dependent signaling, was a unique feature of muscle during aging, type 2 diabetes and obesity (Mathes et al., 2021). *Pim1* knockout could reduce the intramuscular adipose tissue content by inhibiting the adipogenic differentiation of PDGFRα+ mesenchymal progenitors, providing a potential target for the treatment of sarcopenia (Shang et al., 2021). Compared with omental adipose and abdominal subcutaneous tissues, adipocyte progenitor cells were more abundant in gluteofemoral, and their subtypes varied between depots and in patients with type 2 diabetes (Raajendiran et al., 2019). However, to our best knowledge, the exact and complicated function and regulatory mechanism of diverse adipose tissue are still unclear in cattle.

Weighted Gene Co-expression Network Analysis (WGCNA), a biological network analysis method, emerged on the basis of global gene expression patterns aiming to alleviate multiple test problems from extensive data analysis (Langfelder and Horvath, 2008). It can be used to identify modules within a co-expression network, explore the relationship between modules, associate modules with external information (trait, pathway, SNP or QTL), measure the relationship between genes and modules (module membership) or and the studied traits (gene significance). Previously, WGCNA has been widely used to mine genes associated with human disease. In recent years, it has also been utilized to evaluate complex bovine traits and correlate genes and phenotypes in several studies. For instance, ten genes (*PRDX5*, *RAB5C*, *ACTN4*, *SLC25A16*, *MAPK6*, *CD53*, *NCKAP1L*, *ARHGEF2*, *COL9A1*, and *PTPRC*) were detected as hub genes from two critical functional modules associated with mastitis via WGCNA (Ghahramani et al., 2021). Five signifcant functional modules and three hub genes (*GJA1*, *AP2A2*, and *NPAS3*) related to the lactation process were identifed by WGCNA analysis, providing candidate genes to further explore the complex regulatory networks of the lactation process (Farhadian et al., 2021). Kong et al. (Kong et al., 2016) identified a significant module including 764 genes negatively correlated with feed efficiency in a Hereford × Angus population utilizing the WGCNA method. Silva-Vignato et al. (Silva-Vignato et al., 2019) identified three significant modules positively correlated to the backfat thickness, one module negatively correlated with ribeye area and *RSAD2*, *EIF2AK2*, *ACAT1*, and *ACSL1* were considered as hub genes regulating these traits. Furthermore, The gene co-expression network of 5,000 protein-coding genes with majority variations was also constructed across 92 tissues using WGCNA, which is valuable for exploring the molecular mechanisms and elucidating a larger-scale network of functional modules in cattle (Chen et al., 2017).

There is a possibility that WGCNA can also be used to identify the gene networks and hub genes associated with adipose tissues and get a deep understanding of their functions. Hub genes usually interact with many other genes and plays a significant role in regulating biological processes (Yu et al., 2017). Up to now, our study is the first to use WGCNA analysis to explore the gene co-expression network of different adipose tissue types with a sample size of more than 200. In addition, for the genes in the most related module to specific adipose tissue, Kyoto Encyclopedia of Genes and Genomes (KEGG) pathway and gene ontology (GO) analysis were performed to explore their potential functions. Expression analysis for hub genes was conducted to preliminarily verify the accuracy of selection.

## Materials and Methods

### Data Collection and Preprocessing

We downloaded mRNA expression profiles of three studies from the Gene Expression Omnibus (GEO) database (https://www.ncbi.nlm.nih.gov/geoprofiles) (Supplementary Table S1). The first dataset GSE39618 provided a gene expression profile of 35 samples containing four tissue types (intramuscular fat, omental fat, subcutaneous fat and muscle) (Lee et al., 2013). The second one, GSE116775, contained 189 samples including four tissue types (liver, rumen epithelium, subcutaneous fat and muscle) (Sun et al., 2021). Lastly, GSE65125 contained 40 samples including four tissue types (liver, pituitary gland, subcutaneous fat and muscle) (Seo et al., 2016). Supplementary Table S2 provided the detailed experimental and phenotypical information of the three datasets. All the datasets were normalized independently using Robust Multiarray Average (RMA) followed by log2 transformation and quantile normalization.

### Construction of Co-Expression Network

The gene co-expression networks were constructed by the WGCNA package in R (version 4.2.3) (Langfelder and Horvath, 2008). Log transformation was performed on the expression matrix and the normalizeBetweenArrays function of limma (R package) was used for normalization. The top 8,000 genes with the largest median absolute deviation were selected for subsequent analysis. To obtain a scale-free topological network, the pickSoftThreshold function was used to analyze the network topology and choose an appropriate softthresholding power value (β). In this study, the appropriate power value was screened out when the degree of independence reached 0.8. A weighted adjacency matrix was created, defined as A_ij_ = |cor (x_i_, x_j_)|^β^ (i and j represent two different genes, x_i_ and x_j_ are their respective expression values, and A_ij_ represents the Pearson’s correlation coefficient). A one-step approach is adopted to build the network using the blockwiseModules function. The connectivity of a gene in the network is defined as the sum of its adjacency with all other genes. To measure the genes connectivity, the adjacency was transformed into a topological overlap matrix (TOM). Hierarchical clustering was conducted according to TOM-based dissimilarity to allocate genes with similar expression patterns into modules with a minimum cluster size of 50. Highly similar modules were merged with 0.25 as the threshold of cut height.

### Identification of Modules Significantly Associated With Fat Types

Module eigengenes (MEs) were defined as the first principal component of the interested modules, and the expression patterns of genes in the module could be summarized into a single characteristic expression profile. To identify the modules and genes related to fat types, we further associated the modules with phenotypic information. The correlation between MEs and the specific adipose tissue was evaluated by the Pearson’s correlation test with *p* < 0.05 as the cut-off. The module most significantly related to each fat type was considered as the key module and subjected to further analysis.

### Identification of Hub Genes

Gene significance (GS) was defined as the association between gene expression and a specific trait, and could be calculated by the equation GS_i_ = |cor (x_i_, T)|, where x_i_ is the expression of gene i, and T is a sample trait. Meanwhile, module membership (MM) was defined as the correlation between gene expression and each ME, and could be quantified by the equation $MM_{i}^{\text{j}}$ = |cor (x_i_, E^j^)|, where x_i_ is the expression of gene i, and E^j^ is the ME of module j. Genes with GS and MM above a certain threshold in each interested module were selected and considered as potential hub genes. Then the functional protein association networks (PPI) analysis was performed using the STRING website (https://string-db.org/, version 11.5) (Szklarczyk et al., 2021). Moreover, two algorithms (MCC and Degree) of the cytohubba plug-in were used to calculate the core proteins using Cytoscape software (version = 3.8.2) (Doncheva et al., 2019). The overlap of the above results was defined as core genes and the R package of VennDiagram was used to draw the venn diagram.

### Functional Enrichment Analysis

In order to further explore the function of the genes in the module most related to specific adipose types, we performed functional enrichment analysis using the R package clusterProfiler (Yu et al., 2012). The function enrichKEGG and enrichGO were used for the KEGG and GO analysis. The R package org. Bt.eg.db (https://bioconductor.org/packages/release/data/annotation/html/org.Bt.eg.db.html) was used for annotation and conversion of bovine genes. Finally, the top 10 KEGG pathways and top 10 biological process (BP) terms were identified for visualization.

### Expression Analysis of Hub Genes

The hub genes of each adipose tissue (intramuscular fat, omental fat and subcutaneous fat) were further validated by differential expression pattern analysis in the datasets GSE39618, which contained the above three types of fat and muscle. Kruskal-Wallis test was adopted to calculate the difference significance in expression among different groups. The R package ggpubr (https://rpkgs.datanovia.com/ggpubr/index.html) was used for visualization.

### Statistical Analysis

The statistical significance of gene expression in the four tissues was analyzed using non-parametric test or *t* test based on data distribution characteristics. All the analysis were conducted using R software (version 4.2.3) and *p* value <0.05 was considered statistically significant.

## Results

### Construction of Co-Expression Networks

**FIGURE 1:**
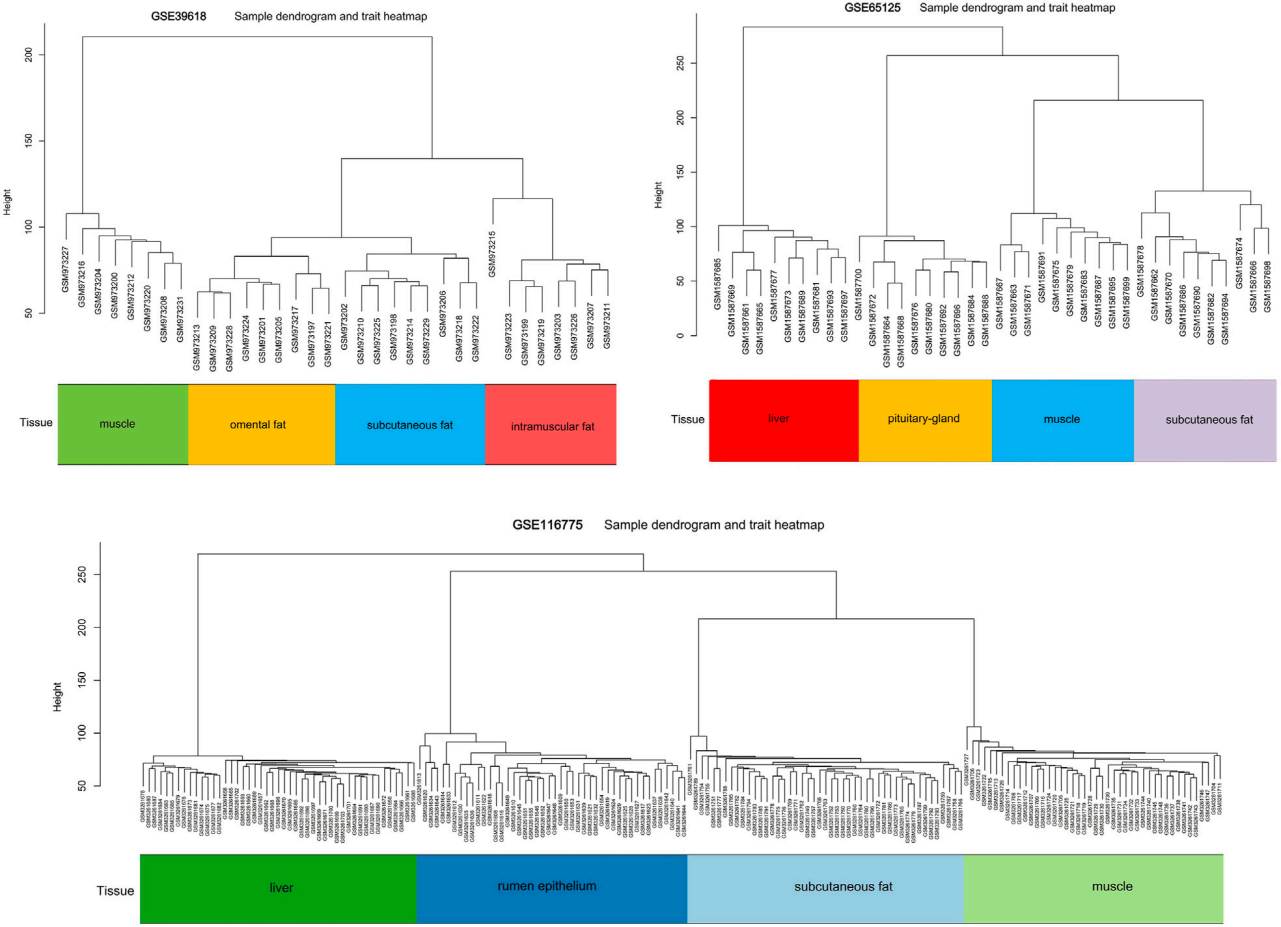
Sample dendrogram and trait heatmap in GSE39618, GSE65125 and GSE116775.

**FIGURE 2:**
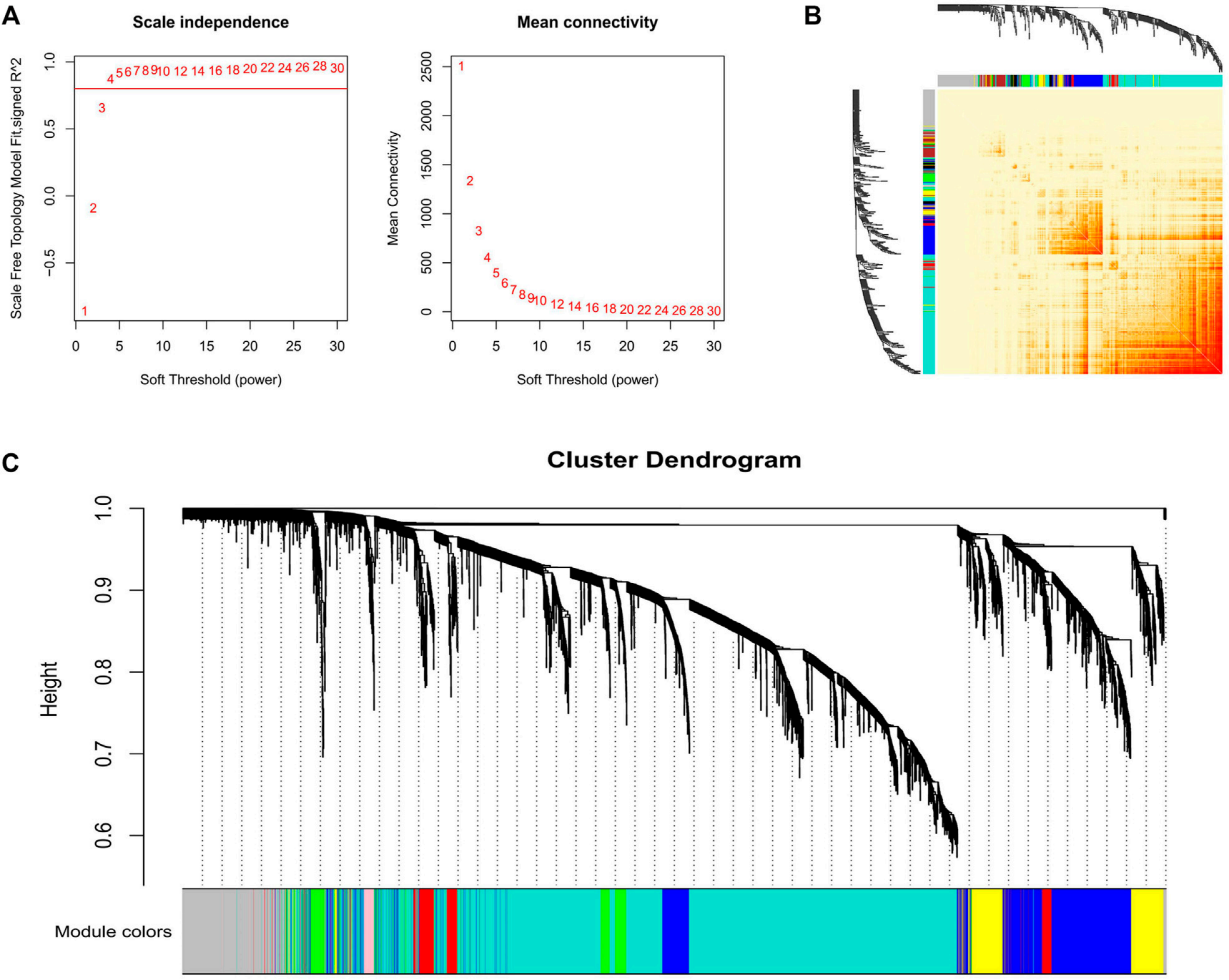
Gene co-expression networks in GSE39618. **(A)** Analysis of the scale-free fit index for various soft-thresholding powers (Left) and analysis of the mean connectivity for various soft-thresholding powers (Right); **(B)** Network heatmap plot in the co-expression modules. **(C)** Clustering dendrogram of the genes.

Three datasets containing adipose tissue were selected for WGCNA analysis in this study. Cluster analysis showed that samples of the same tissue type were classified into a group, indicating it was the main reason (the first principal component) for the difference compared with gender type, breed, feed efficiency, etc. (Figure 1). So these datasets are expected to unearth modules and hub genes specific to adipose tissue type. Subsequently, 0.8 was used as the correlation coefficient threshold to select the soft-thresholding power and construct co-expression networks (Figure 2A, Supplementary Figures S1A, S2A).

Through WGCNA analysis, 25 co-expression modules from the three projects were constructed. In dataset GSE39618, the module comprising most genes was the turquoise one (2,859 genes), followed by the grey module (932 genes), the blue module (897 genes), and the brown module (550 genes) (Figure 2C). In dataset GSE65125, the module comprising most genes was the turquoise one (2,320 genes), followed by the blue module (1,290 genes), the brown module (1,188 genes), and the yellow module (1,076 genes) (Supplementary Figure S1C). In dataset GSE3116775, the module comprising most genes was the turquoise one (1,536 genes), followed by the blue module (1,337 genes), the brown module (1,145 genes), and the yellow module (794 genes) (Supplementary Figure S2C). Network heatmap analysis revealed that genes within modules tend to have higher connectivity and these modules were independent of the others (Figure 2B, Supplementary Figures S1B, S2B).

**FIGURE 3:**
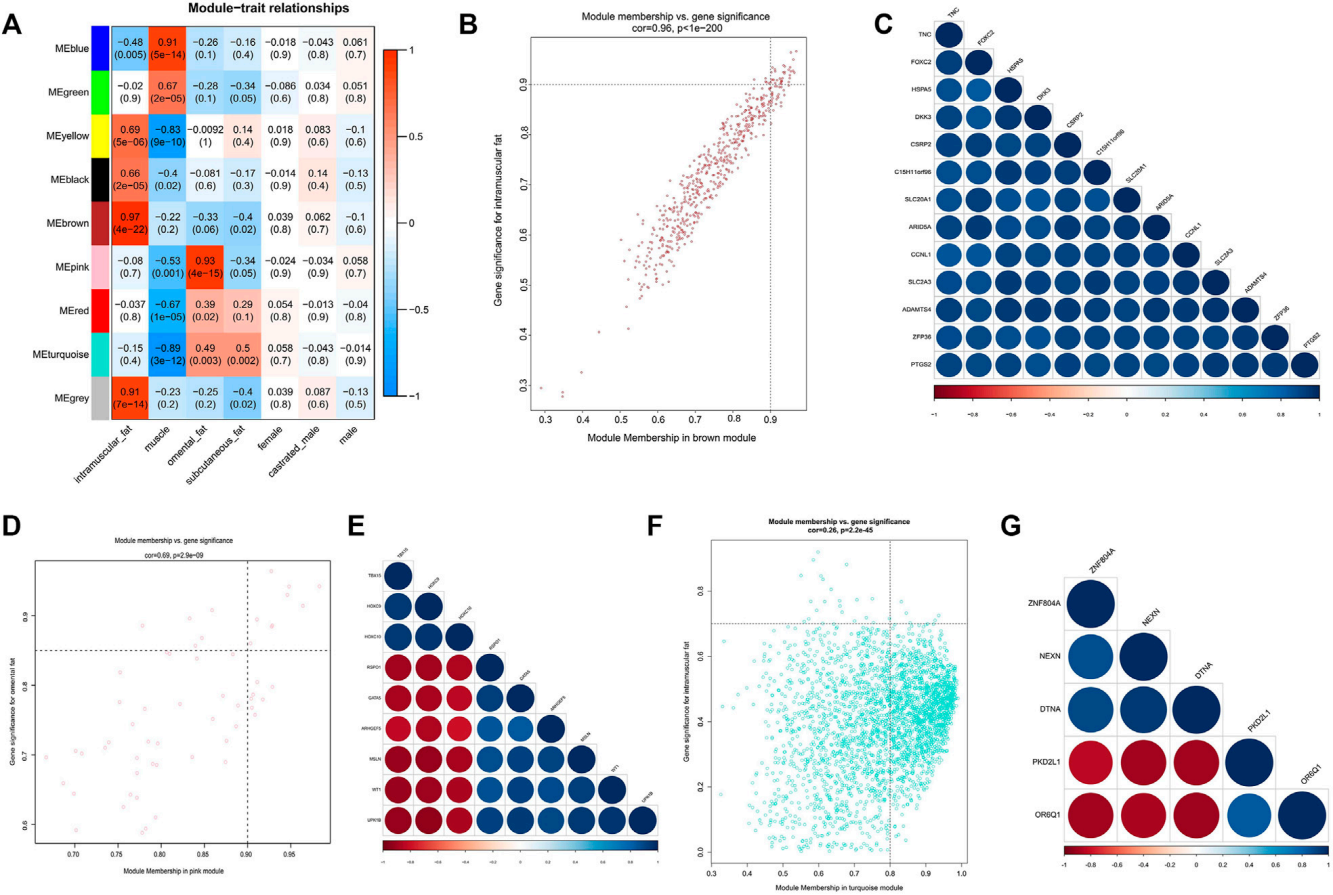
Module-trait correlations analysis of GSE39618. **(A)** Correlation heatmap between modules and trait (correlation coefficient and *p* value are indicated); **(B)** The MM and GS for intramuscular fat in the brown module; **(C)** The correlation relationship of top 13 genes with high MM and GS in the brown module; **(D)** The MM and GS for omental fat in the pink module; **(E)** The correlation relationship of top 9 genes with high MM and GS in the pink module; **(F)** The MM and GS for inter-ribs subcutaneous fat in the turquoise module; **(G)** The correlation relationship of top 5 genes with high MM and GS in the turquoise module.

**FIGURE 4:**
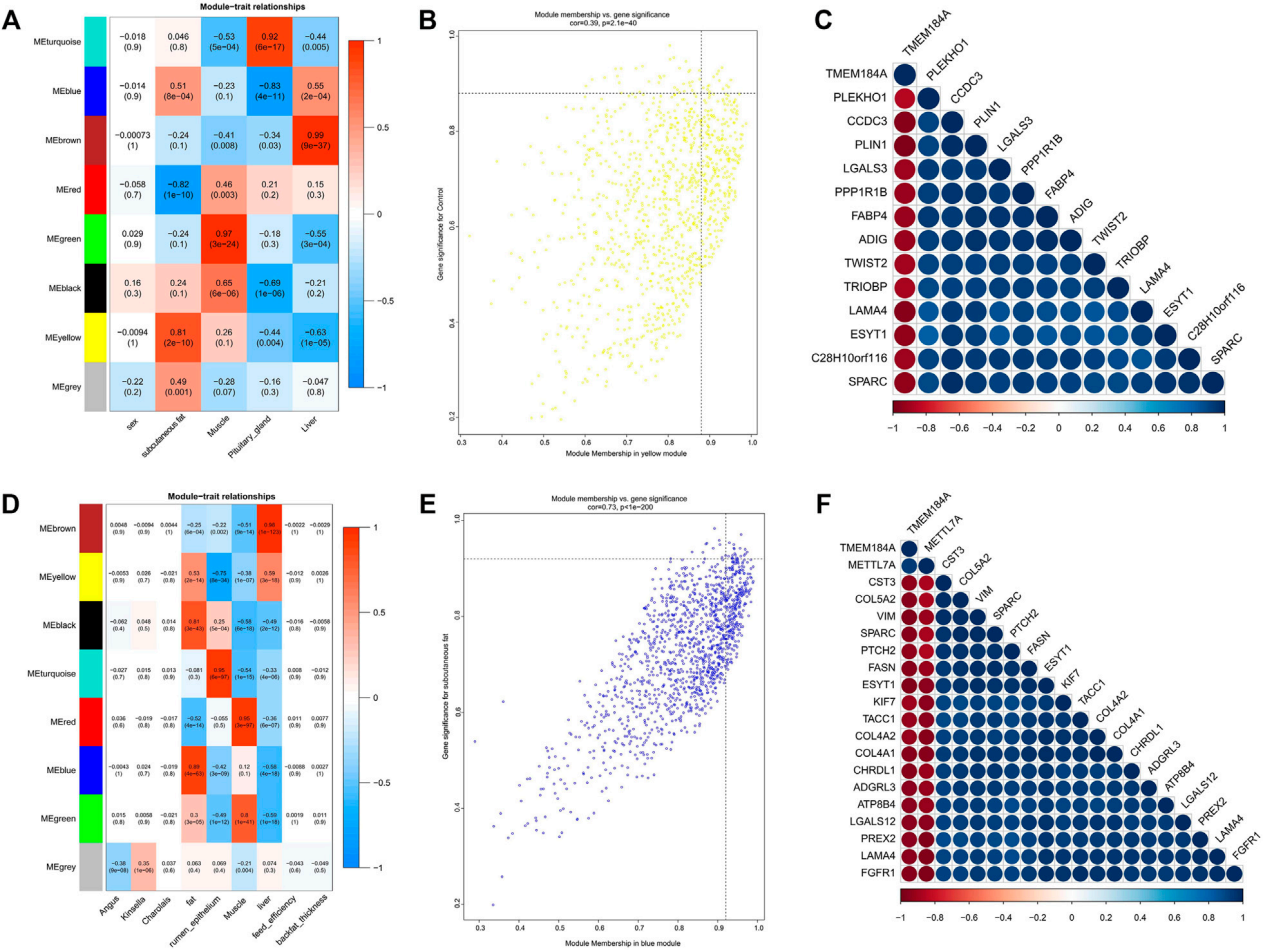
Module-trait correlations analysis of GSE65125 and GSE116775. **(A,D)** Correlation heatmap between modules and trait; **(B)** The MM and GS for abdominal subcutaneous fat in the yellow module; **(C)** The correlation relationship of top 14 genes with high MM and GS in the yellow module; **(E)** The MM and GS for dorsal subcutaneous fat in the blue module; **(F)** The correlation relationship of top 20 genes with high MM and GS in the blue module.

Moreover, we associated the identified modules with external traits and obtained the modules significantly correlated with the traits (tissue types, gender types, breed, feed efficiency or thickness of backfat) (Figures 3A, 4A,D). According to our research purpose, modules significantly related to adipose tissues were selected for subsequent analysis.

### Analysis of Modules Correlated With Intramuscular Adipose Tissue

Module-trait correlation analysis showed that five modules (brown, grey, yellow, black, and blue) were significantly related to intramuscular adipose (Figure 3A). And the brown module showed the most significant association. Figure 3B showed the significance of these genes in the brown module for intramuscular adipose. Notably, some genes such as *TNC*, *FOXC2* and *HSPA5* had high MM values and GS for intramuscular adipose (Figure 3B). Besides, these genes were also closely related to each other (Figure 3C). Thus they were considered as potential hub genes.

**FIGURE 5:**
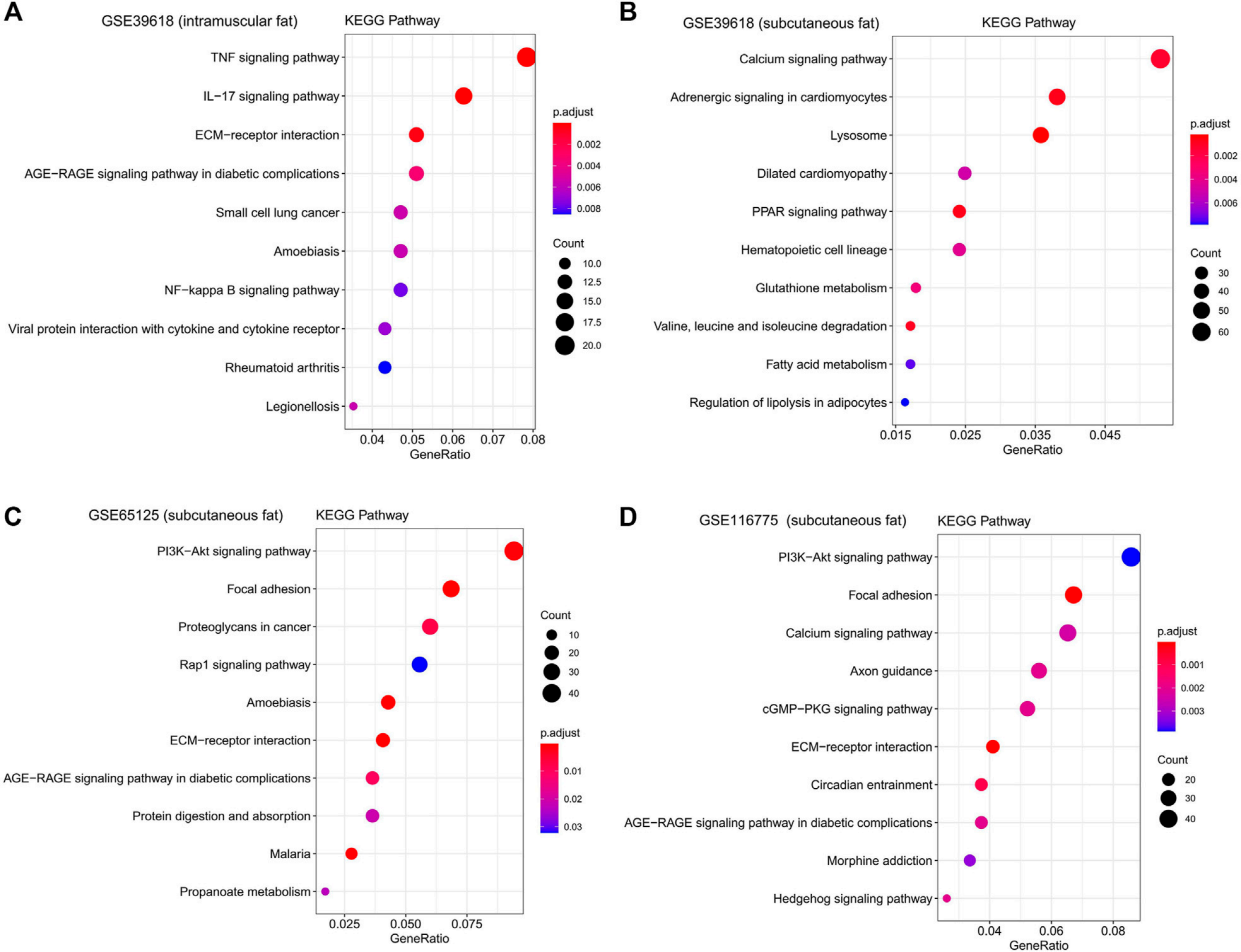
KEGG functional enrichment analysis of interested modules. KEGG analysis of genes significantly related to intramuscular fat in the brown module **(A)**, inter-ribs subcutaneous fat in the turquoise module **(B)**, abdominal subcutaneous fat in the yellow module **(C)** and dorsal subcutaneous fat in the blue module **(D)**.

**FIGURE 6:**
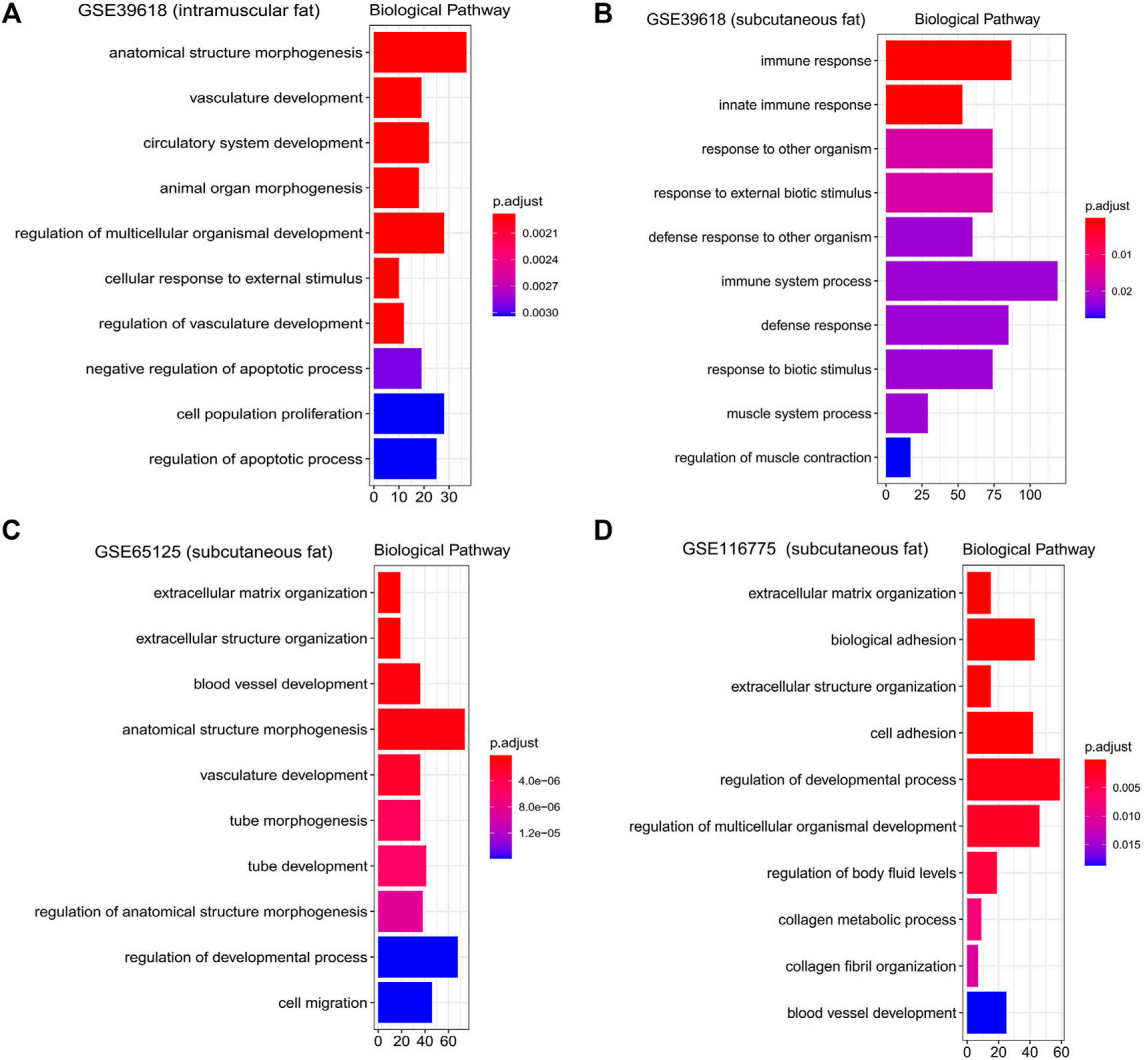
Gene ontology enrichment analysis of interested modules. GO analysis (Biological pathway) of genes significantly related to intramuscular fat in the brown module **(A)**, inter-ribs subcutaneous fat in the turquoise module **(B)**, abdominal subcutaneous fat in the yellow module **(C)** and dorsal subcutaneous fat in the blue module **(D)**.

KEGG functional enrichment analysis showed that the genes in the brown module were mainly enriched in pathways related to inflammation, such as the “TNF signaling pathway,” “IL-17 signaling pathway,” and “NF-kappa B signaling pathway” (Figure 5A). GO functional enrichment analysis revealed that they were mainly enriched in the biological process involved in morphogenesis, development, proliferation, and apoptosis (Figure 6A). Based on the enrichment results, it could be inferred that the intramuscular adipose deposition may be associated with the regulation of inflammation and disease through cell morphogenesis and proliferation, and apoptosis in cattle.

### Analysis of Modules Correlated With Subcutaneous Adipose Tissue

As the three datasets all contained subcutaneous adipose tissue, a total of 11 modules significantly related to subcutaneous adipose were identified. The turquoise module of dataset GSE39678 showed significant association with the subcutaneous fat between the sixth and seventh ribs (Figure 3A). The yellow module (GSE65125) showed the most significant association with the abdominal subcutaneous fat (Figure 4A). The blue module (GSE116775) was most significantly associated with the dorsal subcutaneous fat (Figure 4D). The genes with high MM and GS for inter-ribs (Figure 3F), abdominal (Figure 4B) and dorsal (Figure 4E) subcutaneous fat were screened and exhibited, respectively. And there were also strong correlations among these genes (Figures 3G, 4C,F).

Subsequently, functional enrichment analysis for the three modules was conducted respectively. It was shown that genes of modules related to the above three subcutaneous fat were mainly enriched in pathways related to fat metabolism (such as “PPAR signaling pathway,” “Fatty acid metabolism,” “PI3K-Akt signaling pathway,” and “Hedgehog signaling pathway”), disease (such as “Dilated cardiomyopathy,” “AGE−RAGE signaling pathway in diabetic complications,” and “Proteoglycans in cancer”) and cell communication (such as “Calcium signaling pathway,” “Focal adhesion,” and “ECM-receptor interaction”) (Figures 5B–D). GO functional enrichment analysis revealed that they were mainly enriched in biological processes related to immune response (such as “innate immune response,” “immune system process,” and “response to biotic stimulus”), cellular organization and development (such as “extracellular matrix organization,” “regulation of developmental process”), and signal transduction (such as “biological adhesion,” “cell adhesion”) (Figures 6B–D).

Furthermore, the intersection of genes in the modules significantly related to the three adipose tissues was obtained (Figure 8A), and the KEGG and GO enrichment was consistent with the above results (Figures 8B,C). This provides a basis for the study of subcutaneous adipose tissue as an endocrine organ to regulate the occurrence of diseases in cattle.

### Analysis of Modules Correlated With Omental Adipose Tissue

Three modules significantly related to omental adipose were identified by module-trait correlations analyssis (Figure 3A). It clearly indicated that the pink module was most significantly associated with omental adipose. Figure 3D showed the significance of these genes in the pink module. Notably, several genes in the pink module such as *WT1*, *ARHGEF5*, and *HOXC9* had high MM and GS for omental adipose. In addition, they were highly correlated with each other and considered as potential hub genes.

KEGG functional enrichment analysis revealed that the genes in the pink module were mainly enriched in pathways regulating fat metabolism (such as “Wnt signaling pathway” and “Hippo signaling pathway”), cell proliferation and cancer (such as “Transcriptional misregulation in cancer” and “Basal cell carcinoma”), and signal transduction (such as “Axon guidance” and “Viral protein interaction with cytokine and cytokine receptor”) (Supplementary Figure S3). GO functional enrichment analysis revealed that they were mainly enriched in the biological process being involved in tissue morphogenesis and development, and cell proliferation and adhesion (Supplementary Figure S3). However, the *p*-value of enrichment analysis was not significant, which may be due to the deviation caused by the small number of genes in this module.

### Identification and Analysis of Hub Genes in Intramuscular Adipose Tissue

**FIGURE 7:**
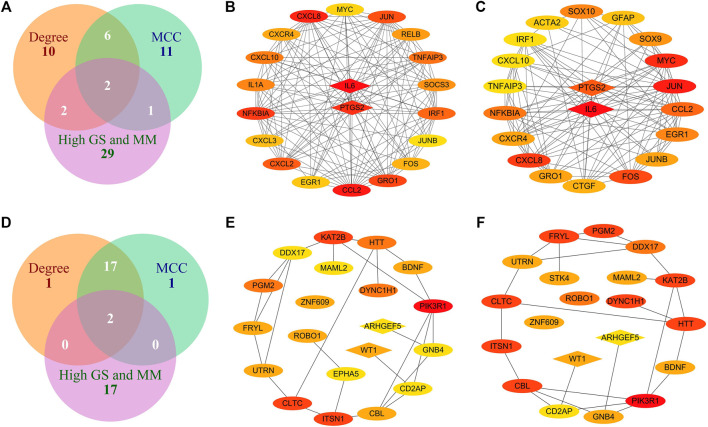
Identification of hub genes in intramuscular and omental fat. **(A)** Intersection of genes with high MM and GS for intramuscular fat and the top 20 hub genes identified by MCC and Degree algorithms in the brown module; **(B–C)** Top 20 hub genes identified by MCC **(B)** and Degree **(C)** algorithms in the brown module; **(D)** Intersection of genes with high MM and GS for omental fat and the top 20 hub genes identified by MCC and Degree algorithms in the pink module; **(E–F)** Top 20 hub genes identified by MCC **(E)** and Degree **(F)** algorithms in the pink module (the redder color indicates the higher interaction score).

We obtained 34 potential hub genes under the condition of MM > 0.95 and GS > 0.95 in the brown module (Figure 3B). Simultaneously, all the 550 genes of this module were sent to STRING to get a PPI network and the top 20 hub genes were identified by MCC and Degree algorithms (Figures 7B,C). *PTGS2* and *IL6* were confirmed as the final key genes by the intersection of 61 potential hub genes and the top 20 genes in the PPI network. Furthermore, the expression of *PTGS2* and *IL6* was significantly increased (all *p* < 0.01) in intramuscular fat compared to omental fat, subcutaneous fat and muscle (Figure 10A).

### Identification and Analysis of Hub Genes in Subcutaneous Adipose Tissue

**FIGURE 8:**
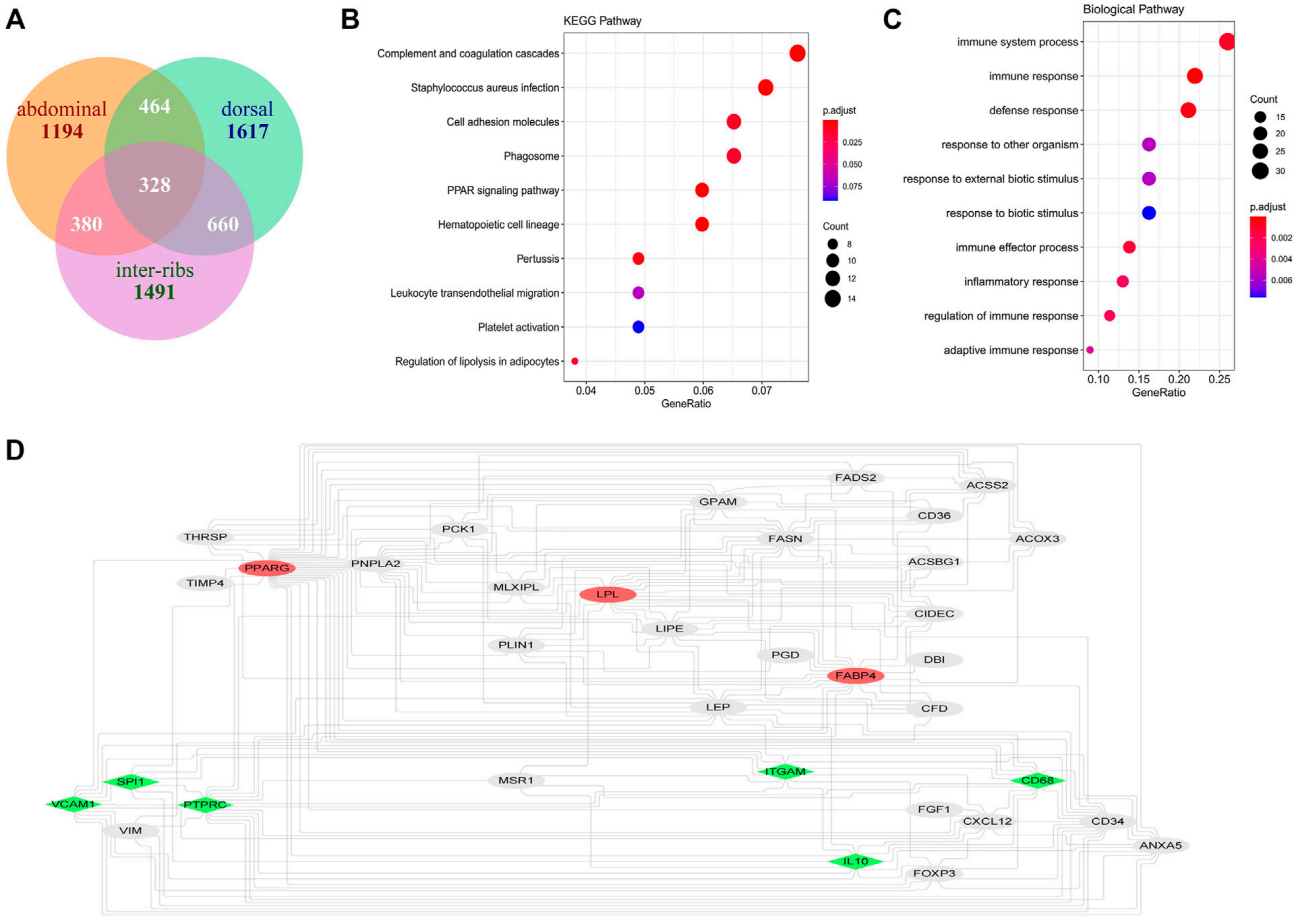
Identification of hub genes in subcutaneous fat. **(A)** Intersection of genes significantly related to inter-ribs, abdominal and dorsal subcutaneous fat; **(B)** KEGG analysis of the 328 shared genes of the three subcutaneous fat; **(C)** Biological pathway analysis of the 328 shared genes of the three subcutaneous fat; **(D)** Identification of the common hub genes of the three subcutaneous fat (the red ones are the seed genes, and the green ones are the hub genes that both interact with the seeds calculated by MCC and Degree algorithms).

The potential hub genes of inter-ribs (Figure 3F), abdominal (Figure 4B) and dorsal (Figure 4E) subcutaneous fat were obtained with high MM and GS values. Since there was no overlap in these potential hub genes, the intersection of genes in the modules significantly related to the three adipose tissues was used to identify the hub genes by PPI analysis (Figure 8A). Firstly, *PPARG*, *FABP4* and *LPL*, the three marker genes of adipogenic differentiation, were used as seeds to screen networks that interacted with them. Secondly, the 328 overlapped genes were used to identify the top 20 hub genes by MCC and Degree algorithms. Finally, the overlaps were confirmed as the key genes (*CD68*, *SPI1*, *PTPRC*, *IL10*, *VCAM1* and *ITGAM*).

**FIGURE 9:**
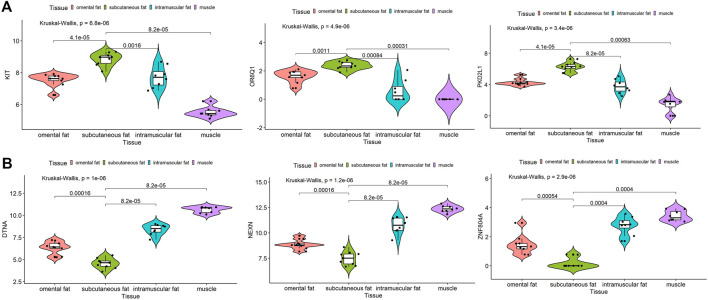
Expression analysis of hub genes in subcutaneous fat. Expression levels of *KIT*, *OR6Q1* and *PKD2L1* **(A)** were significantly increased and *DTNA*, *NEXN* and *ZNF804A* **(B)** were significantly decreased in subcutaneous fat.

Furthermore, both the hub gene of inter-ribs subcutaneous fat and the shared hub genes of three subcutaneous fat tissues were analyzed by expression in dataset GSE39618. Results showed that the expression of *KIT*, *OR6Q1* and *PKD2L1* was significantly increased (*p* < 0.01), and *DTNA*, *NEXN* and *ZNF804A* was significantly decreased (*p* < 0.01) in subcutaneous fat compared to omental fat, intramuscular fat and muscle (Figure 9A). The expression of *IL10* and *VCAM1* was not different among the three types of subcutaneous fat, but was significantly higher than that of muscle (*p* < 0.01) (Supplementary Figure S4A). The expression of *PTPRC* and *SPI1* was significantly higher than that of intramuscular fat and muscle (*p* < 0.01), but there was no difference with omental fat (Supplementary Figure S4B).

### Identification and Analysis of Hub Genes in Omental Adipose Tissue

**FIGURE 10:**
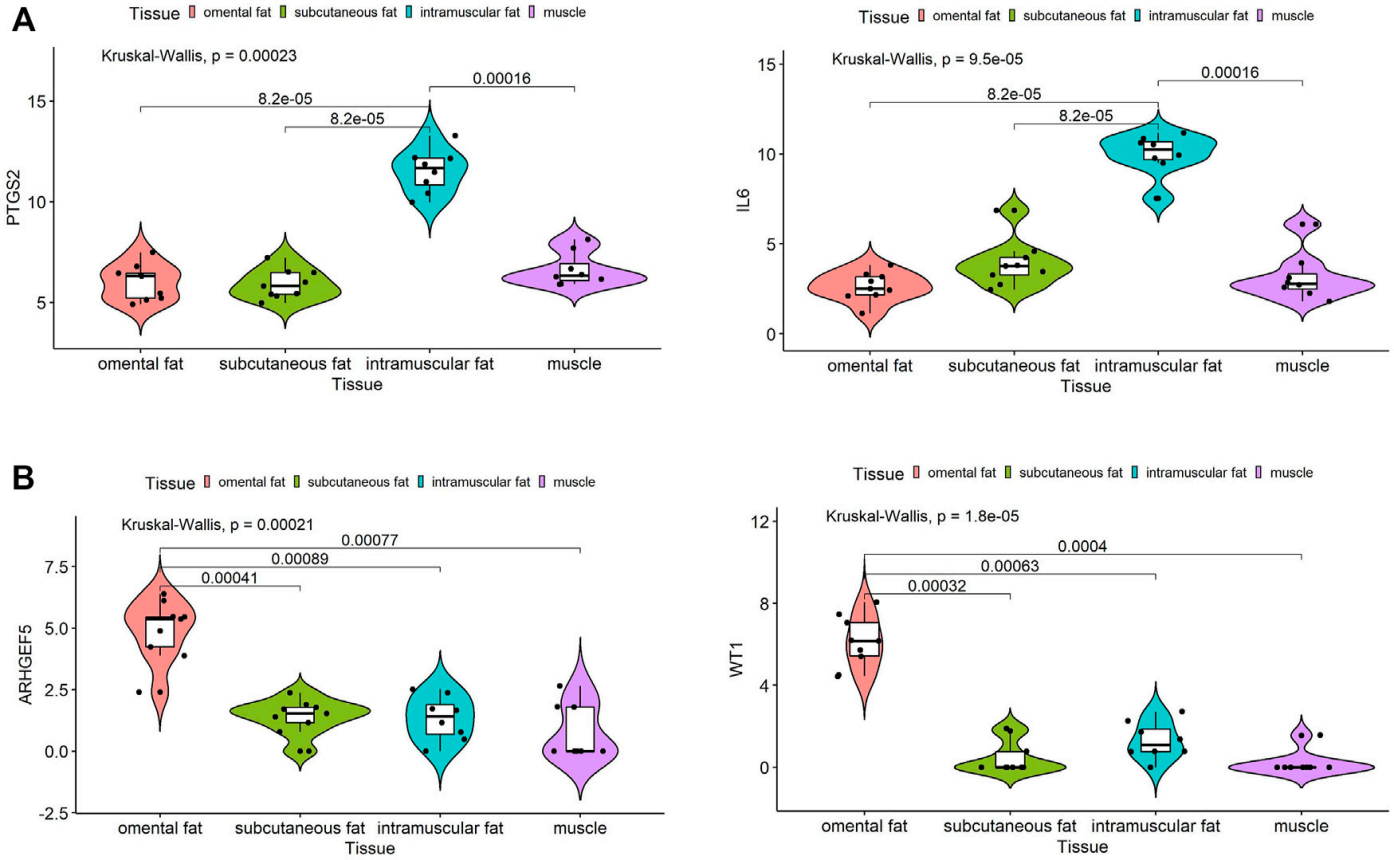
Expression analysis of hub genes in intramuscular and omental fat. Expression levels of *PTGS2* and *IL6* **(A)** were significantly increased in intramuscular fat, and *WT1* and *ARHGEF5* **(B)** were significantly increased in omental fat.

When the criteria was set as MM > 0.8 and GS > 0.8, 19 potential hub genes were obtained in the pink module (Figure 3D). Simultaneously, all the 57 genes of this module were sent to STRING to get a PPI network and the top 20 hub genes were identified by MCC and Degree algorithms (Figures 7E,F). *WT1* and *ARHGEF5* were confirmed as the final key genes by the intersection of the potential hub genes and the top 20 genes in the PPI network. Furthermore, the expression of *WT1* and *ARHGEF5* was significantly increased (*p* < 0.01) in omental fat compared to intramuscular fat, subcutaneous fat and muscle (Figure 10B).

## Discussion

Backfat thickness and beef marbling are two critical indexes for bovine breeding, which mainly depend on the dorsal subcutaneous fat and intramuscular fat content (Cameron et al., 1994; Taniguchi et al., 2008; Zhang et al., 2016; Lee et al., 2019). Omental fat, as visceral fat, might be related to the occurrence of various diseases (Donkers et al., 2021; Favre et al., 2021; Verduin et al., 2021). It is of great significance for bovine breeding to obtain core genes by analyzing the molecular regulatory network of the above three adipose tissues. As far as we know, our study is the first one to build the adipose tissue-related gene network with samples more than 200 using WGCNA. Several gene co-expression modules associated with intramuscular, subcutaneous and omental fat were identified and hub genes were also obtained, presenting new insights into the diversed function and regulatory mechanism of bovine adipose tissue.

In order to verify the accuracy of module identification, we further searched the adipogenic marker gene (*PPARG*, *FABP4*, and *LPL*) in the three datasets and determined the modules that they located in. Results showed that they were in turquoise module of GSE39618, yellow module of GSE65125, and blue module of GSE116775, which were all most significantly correlated with the subcutaneous adipose tissue. It suggested that lipid metabolism might be more active in subcutaneous fat compared with intramuscular fat, omental fat, liver, rumen epithelium, etc. Furthermore, We compared the identified modules and genes with previous studies. For instance, the three key transcription factors (*PPARGC1A*, *HNF4G*, and *FOXP3*) in turquoise module (Supplementary Table S3) that correlated with subcutaneous fat were also proved to regulate carcass IMF of beef cattle through the previous genomewide association studies (GWAS) (Ramayo-Caldas et al., 2014a; Ramayo-Caldas et al., 2014b). The study on gene expression profile of musculus longissimus dorsi in bulls identified 32 differentially expressed genes between high IMF (7.0%) and low IMF (1.9%) (Komolka et al., 2016), among which *CILP2* and *SCD5* in the brown module, *SLC43A2* and *INSIG1* in the blue module, and *IL4R* and *SESN1* in the yellow module were also significantly associated with intramuscular fat in our study (Figure 3; Supplementary Table S3).

Hub genes were defined based on the connectivity with other genes and correlation between gene expression and a specific trait (Panahi et al., 2020). Therefore, they may have a signifcant biological function in regulating the trait. In our study, *PTGS2* and *IL6* were determined as hub genes in intramuscular fat, which could be used to distinguish it from omental fat, subcutaneous fat and muscle. *PTGS2* (prostaglandin-endoperoxide synthase 1, also known as *COX-2*) is involved in the pathways of “Regulation of lipolysis in adipocytes” (Rouzer and Marnett, 2009). It participants the metabolism of arachidonic acid by catalyzing oxaloacetic acid (OAA) to produce prostaglandin E2 (PGE2), which stimulates the production of leptin through EP3, thus promoting adipogenesis and inhibiting lipolysis to promote obesity (Jaworski et al., 2009). In mice, the over-expression of *COX-2* in white adipose tissue has been shown to induce *de novo* recruitment of brown adipose tissue, and then facilitate systemic energy expenditure to protect against high-fat diet-induced obesity (Li et al., 2010). In human, *COX-2* was highly expressed in the subcutaneous adipose tissue of obese individuals, and the administration of PGE2 and arachidonic acid could stimulate the release of leptin in the adipose tissue (Chan et al., 2016).

*IL6* (interleukin 6) was known as a marker gene for inflammation (Fonseca et al., 2009). In the recent years, it was also found to regulate fat metabolism and related diseases through several pathways such as “Insulin resistance” (Xuguang et al., 2019), “Non-alcoholic fatty liver disease” (Fang et al., 2019), “AGE-RAGE signaling pathway in diabetic complications” (Abo et al., 2020), “Lipid and atherosclerosis” (Ridker et al., 2018) and “PI3K-Akt signaling pathway” (Fajgenbaum et al., 2019). For instance, in “Non-alcoholic fatty liver disease” pathway, IL6 activates its receptor IL6R and regulates the activity of lipogenic enzymes through SOCS3 and SREBP-1c to influence *de novo* fatty acid synthesis. Meanwhile, the concentrations of IL-6 and TNFα in serum were generally increased in obese individuals, and these cytokines could effect the expression of *COX-2* to promote PGE2 production, thus regulating the process of adipogenesis.

*WT1* and *ARHGEF5* could be used to distinguish omental fat from intramuscular fat, subcutaneous fat and muscle, and are considered as two key genes. *WT1* (Wilms tumor protein 1) plays multiple roles in development, tissue homeostasis and disease (Hastie, 2017). It is also a marker gene of visceral adipocyte precursor, which expressed in small proliferative adipocytes (SPA) isolated from epididymal SPA, but not inguinal SPA (Taguchi et al., 2020). During the late period of mouse gestation, most visceral white adipose tissue (WAT) but no subcutaneous WAT arises from cells expressing *Wt1* (Chau and Hastie, 2015). However, the expression of *Wt1* showed an increase in subcutaneous adipose tissue after a short period of fasting, and decreased sharply as the fasting progress (Tang et al., 2017). Moreover, the *Wt1*-positive adipocytes tend to have fewer, larger lipid droplets than the *Wt1*-negative lineage. Except as a marker gene, it could also be used to recognize visceral WAT identity and the progenitor population, permitting further analysis of different cell populations (Cleal and Chau, 2016). In cattle, *WT1* isoforms were found to regulate steroidogenesis by modulating the PI3K/AKT and ERK1/2 pathways in granulosa cells (Meng et al., 2019). In our study, *WT1* was determined as a key gene of omental fat (a kind of visceral fat), indicating that it might also play an essential role in bovine visceral fat as well.

As another key gene identified in omental fat, the research on Rho guanine nucleotide exchange factor 5 (*ARHGEF5*) has focused on the regulation of disease and cell junction by now. For instance, *ARHGEF5* localizes to the neuromuscular junctions (NMJs) and binds α-Dystrobrevin 1 to regulate the integrity of NMJs in mice (Bernadzki et al., 2020). ARHGEF5 formed a ternary complex with Src and phosphoinositide 3-kinase and played crucial roles in Src-induced podosome formation, which may be involved in cancer malignancy invasion (Kuroiwa et al., 2011). Furthermore, ARHGEF5 had the potential to promote tumor proliferation via the phosphatidylinositol 3-kinase (PI3K) pathway (Komiya et al., 2016). Previous studies have confirmed that PI3K signaling pathway is involved in the regulation of fat metabolism, inflammation and cancers (Porta et al., 2014; Xia and Xu, 2015; Koundouros and Poulogiannis, 2020). Although the exact function of *ARHGEF5* in fat metabolism has not been studied, *ARHGEF2*, the other member of *ARHGEF* family, was found to be associated with intramuscular fatty acid composition in porcine (Ramayo-Caldas and Ballester et al., 2014) and childhood obesity (Zhu et al., 2018). In this sdudy, we identified *ARHGEF5* as the key gene of bovine omental fat, which laid a foundation for studying its function in visceral fat.

Adipose tissues from different anatomical locations usually differ in gene expression and regulatory mechanisms (Schweizer et al., 2020; Silva and Baptista, 2019). According to the anatomical location, subcutaneous adipose tissues also vary in their gene expression and function (Martos-Moreno et al., 2013). Since the three subcutaneous adipose tissues in this study came from different anatomical locations, we analyzed them and excavated the key genes of inter-ribs (*ZNF804A*, *NEXN*, *DTNA*, etc.), abdominal (*PLIN1*, *FABP4*, *ADIG*, etc.), and dorsal (*CST3*, *FASN*, *KLF7*, etc.) subcutaneous fat (Figures 3G, 4C,F). Expression analysis of inter-ribs subcutaneous fat showed that three key genes (*KIT*, *OR6Q1* and *PKD2L1*) were up-regulated and three (*DTNA*, *NEXN* and *ZNF804A*) were down-regulated. Two common key genes (*IL10* and *VCAM1*) obtained from the three subcutaneous adipose tissues could distinguish fat from muscle, but their expression did not differ among intramuscular, subcutaneous and omental adipose tissues. Another two common key genes (*PTPRC* and *SPI1*) showed a significantly higher expression than that of intramuscular fat and muscle, rather than omental fat. These results were consistent with the sample expression clustering (Figure 1), indicating that the gene expression patterns of inter-ribs subcutaneous fat and omental fat were the most similar. Subcutaneous fat from different anatomical positions may located in various microenvironments, resulting in varied metabolic functions and regulatory mechanisms (Quail and Dannenberg, 2019; Rajbhandari et al., 2019). The selection of key genes by intersection may disguise the difference of specific subcutaneous fat, which is also the reason that they were unable to distinguish different types of adipose tissue.

Although our study is the first to investigate the co-expression gene networks associated with types of adipose tissue with a large sample size, it also has limitations. On one hand, we did not further study the exact mechanism of the identified hub genes. On the other hand, we used the data from three different studies in the WGCNA analysis and identification of hub genes. These studies contained samples of distinct breed, age, sex, tissue types, nutrition level, etc. Fortunately, these factors showed much less influence on gene expression compared with tissue types (Figure 1), so it was speculated that they had little effect on the overall analysis results.

In summary, we constructed a gene co-expression network related to three adipose types using 264 bovine tissue samples by WGCNA analysis. Our study identified 19 modules that significantly associated with adipose tissue types and analyzed the functional biological pathways of genes in the interested modules. Furthermore, we summarized the signaling pathways involved in genes significantly associated with adipose tissue types in a graphical abstract (Supplementary Figure S5). At last, key genes for intramuscular fat (*PTGS2* and *IL6*), omental fat (*ARHGEF5* and *WT1*), and subcutaneous fat (*KIT*, *QR6Q1*, *PKD2L1*, etc.) were obtained and verified. Meanwhile, *IL10* and *VCAM1* have a potential to distinguish adipose and muscle. These findings provide new insights into the function of adipose tissue and laid foundation for further exploration of the exact molecular mechanism of hub genes and functional pathways in cattle.

## Data Availability

Publicly available datasets were analyzed in this study. This data can be found here: The datasets used in the study are stored in the Gene Expression Omnibus (GEO) database. Below are the direct links (GSE39618:https://www.ncbi.nlm.nih.gov/geo/query/acc.cgi?acc = GSE39618; GSE116775:https://www.ncbi.nlm.nih.gov/geo/query/acc.cgi?acc = GSE116775; GSE65125:https://www.ncbi.nlm.nih.gov/geo/query/acc.cgi?acc = GSE65125).
