# Supplementary figures and images for: Identifying Key Genes and Functionally Enriched Pathways of Diverse Adipose Tissue Types in Cattle
Source: Front Genet. 2022 Feb 14;13:790690. doi: 10.3389/fgene.2022.790690 (PMC8884536; doi:10.3389/fgene.2022.790690)

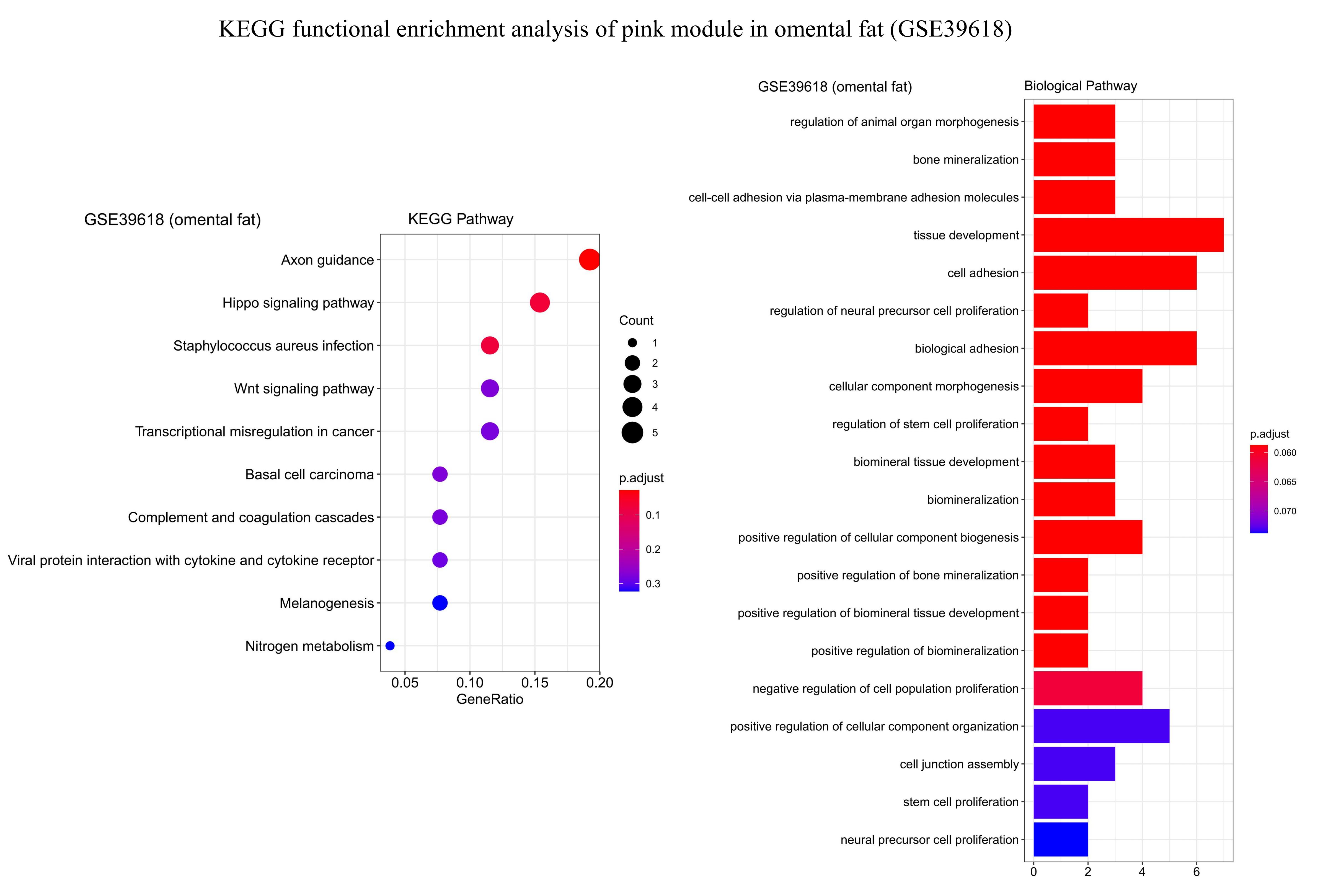

Supplement: Supplementary file 2 [file Image3.JPEG]

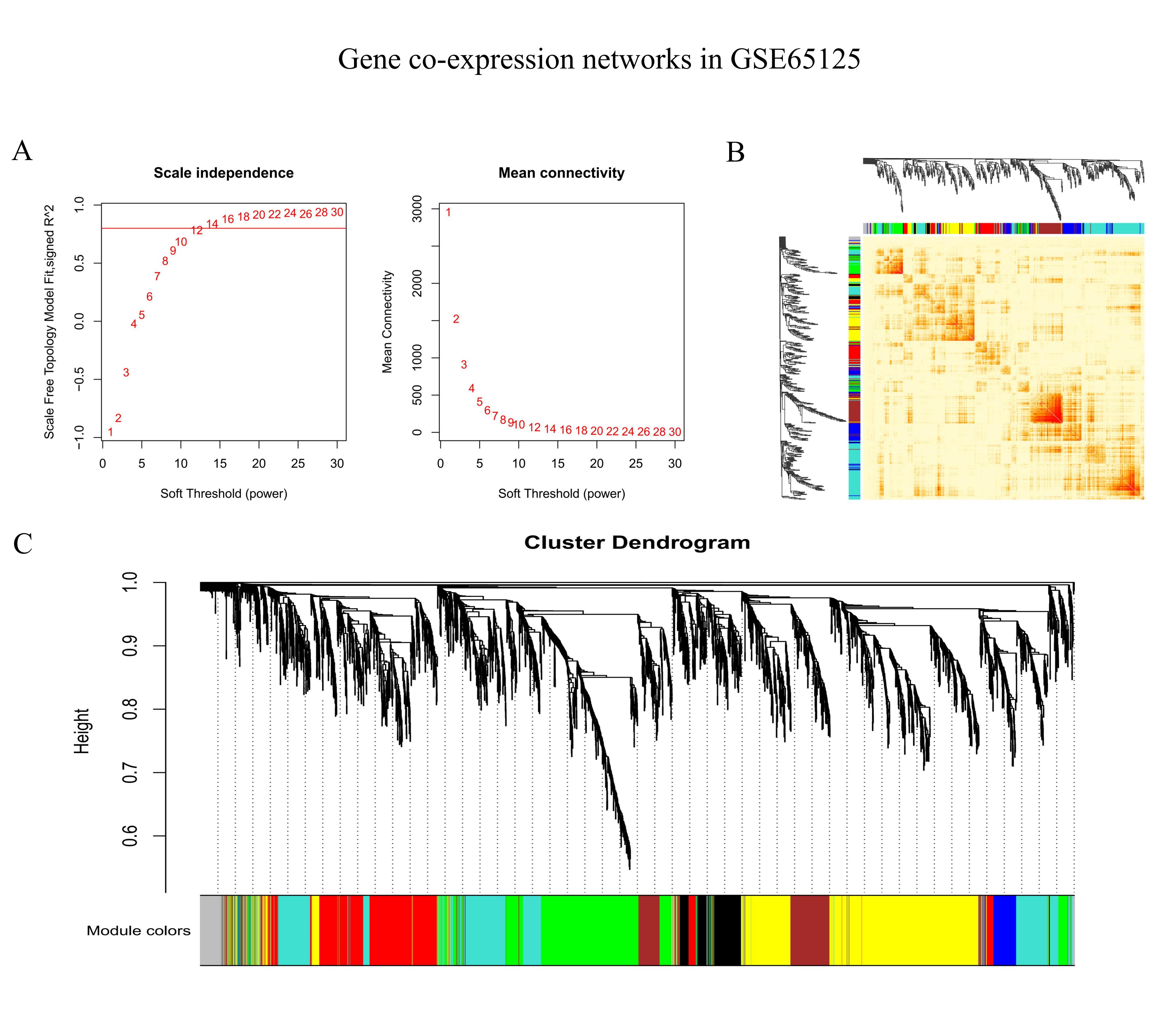

Supplement: Supplementary file 4 [file Image1.JPEG]

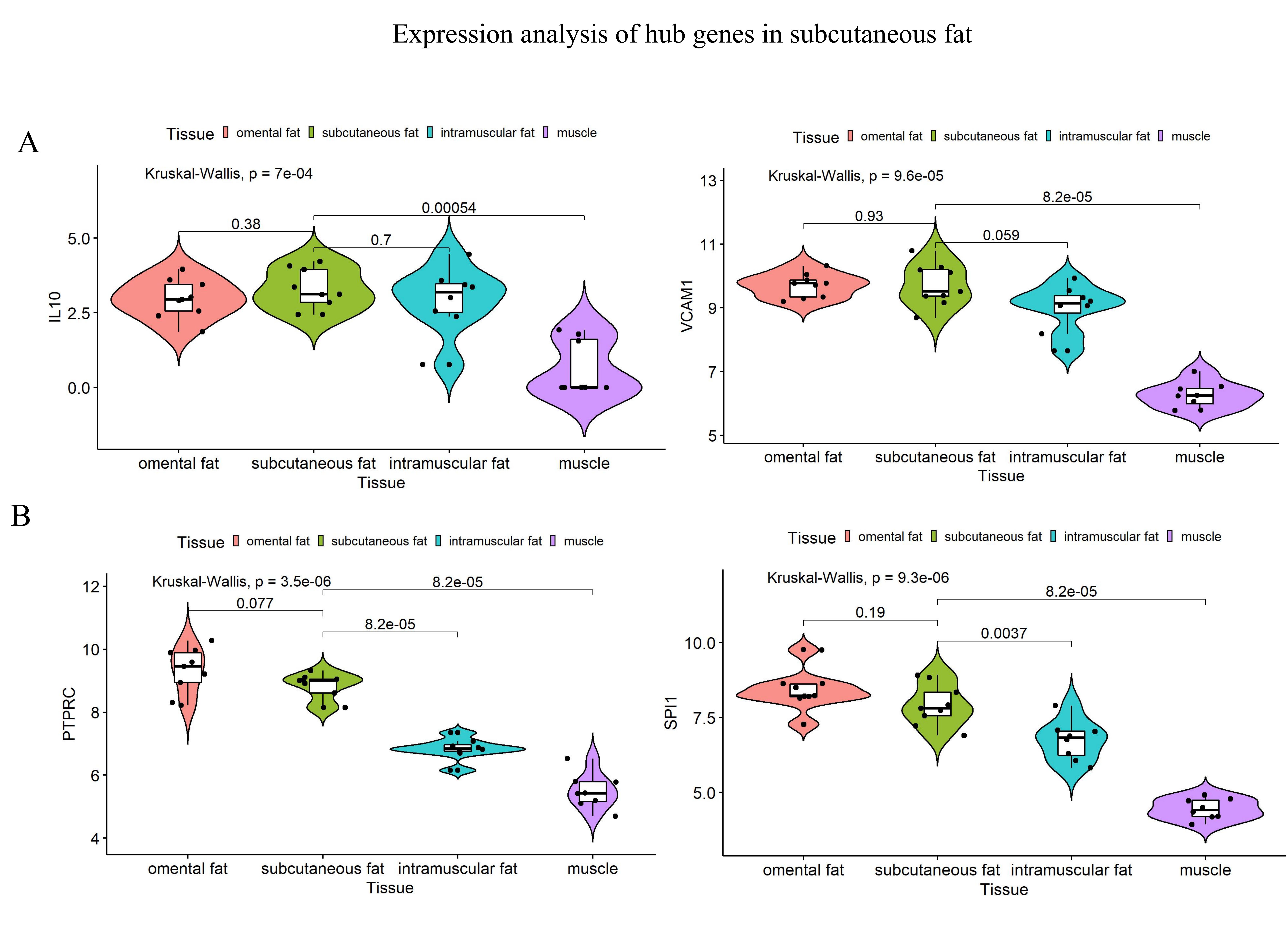

Supplement: Supplementary file 5 [file Image4.JPEG]

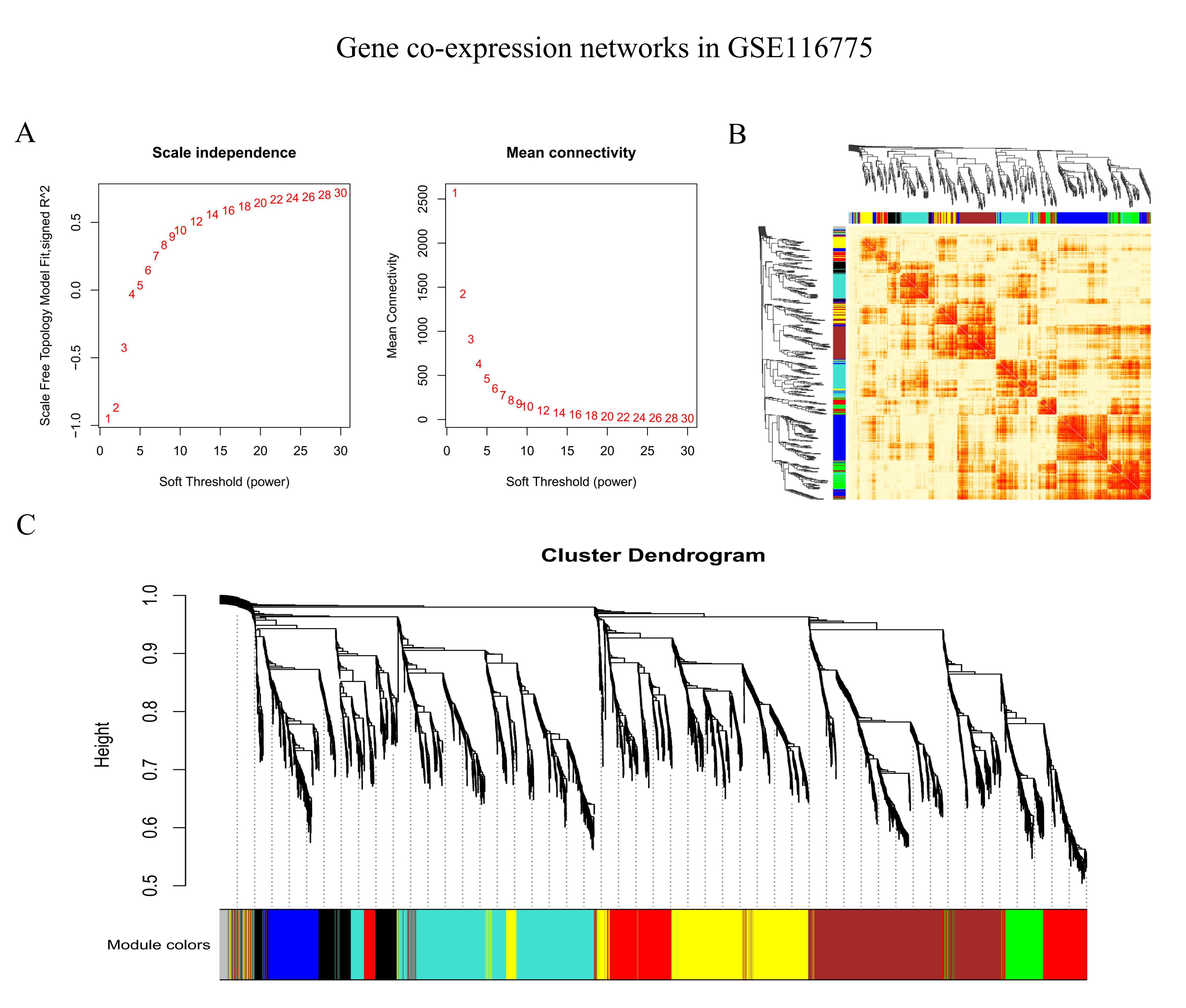

Supplement: Supplementary file 6 [file Image2.JPEG]

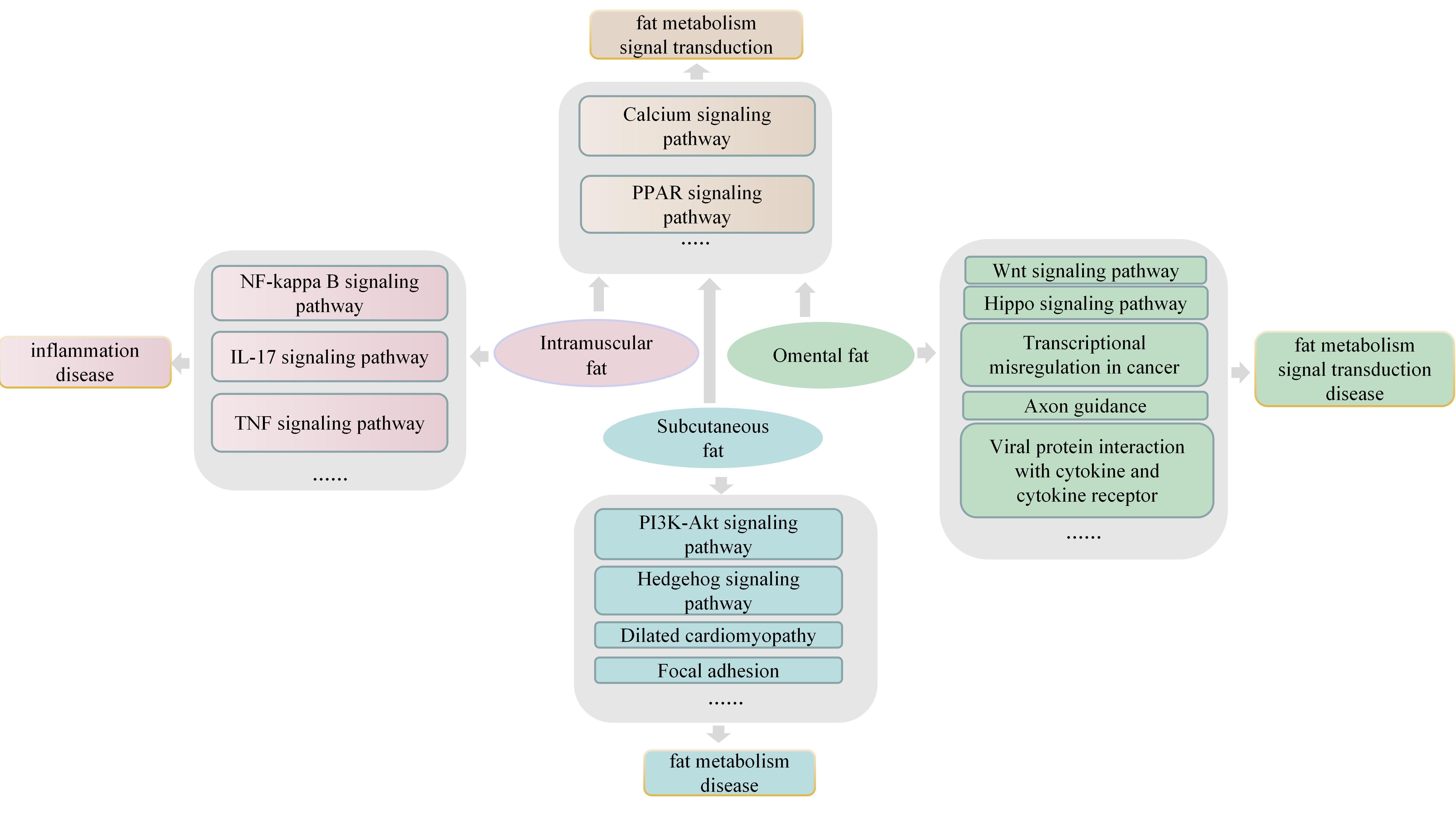

Supplement: Supplementary file 7 [file Image5.JPEG]
